# Treatment of Factor-Xa Inhibitor-associated Bleeding with Andexanet Alfa or 4 Factor PCC: A Multicenter Feasibility Retrospective Study

**DOI:** 10.5811/westjem.60587

**Published:** 2023-08-22

**Authors:** Adam J. Singer, Mauricio Concha, James Williams, Caitlin S. Brown, Rafael Fernandes, Henry C. Thode, Marylin Kirchman, Alejandro A. Rabinstein

**Affiliations:** * Renaissance School of Medicine at Stony Brook University, Department of Emergency Medicine, Stony Brook, New York; † Sarasota Memorial Hospital, Department of Neuroscience, Sarasota, Florida; ‡ Meritus Health, Department of Emergency Medicine, Hagerstown, Maryland; § Mayo Clinic-Rochester, Department of Pharmacy, Rochester, Minnesota; ∥ Mayo Clinic-Rochester, Department of Neurosurgery, Rochester, Minnesota

## Abstract

**Background:**

There are no randomized trials comparing andexanet alfa and 4 factor prothrombin complex concentrate (4F-PCC) for the treatment of factor Xa inhibitor (FXa-I)-associated bleeds, and observational studies lack important patient characteristics. We pursued this study to demonstrate the feasibility of acquiring relevant patient characteristics from electronic health records. Secondarily, we explored outcomes in patients with life-threatening FXa-I associated bleeds after adjusting for these variables.

**Methods:**

We conducted a multicenter, chart review of 100 consecutive adult patients with FXa-I associated intracerebral hemorrhage (50) or gastrointestinal bleeding (50) treated with andexanet alfa or 4F-PCC. We collected demographic, clinical, laboratory, and imaging data including time from last factor FXa-I dose and bleed onset.

**Results:**

Mean (SD) age was 75 (12) years; 34% were female. Estimated time from last FXa-I dose to bleed onset was present in most cases (76%), and patients treated with andexanet alfa and 4F-PCC were similar in baseline characteristics. Hemostatic efficacy was excellent/good in 88% and 76% of patients treated with andexanet alfa and 4F-PCC, respectively (*P* = 0.29). Rates of thrombotic events within 90 days were 14% and 16% in andexanet alfa and 4F-PCC patients, respectively (*P* = 0.80). Survival to hospital discharge was 92% and 76% in andexanet alfa and 4F-PCC patients, respectively (*P* = 0.25). Inclusion of an exploratory propensity score and treatment in a logistic regression model resulted in an odds ratio in favor of andexanet alfa of 2.01 (95% confidence interval 0.67–6.06) for excellent/good hemostatic efficacy, although the difference was not statistically significant.

**Conclusion:**

Important patient characteristics are often documented supporting the feasibility of a large observational study comparing real-life outcomes in patients with FXa-I-associated bleeds treated with andexanet alfa or 4F-PCC. The small sample size in the current study precluded definitive conclusions regarding the safety and efficacy of andexanet alfa or 4F-PCC in FXa-I-associated bleeds.

## INTRODUCTION

The use of factor Xa inhibitors (FXa-I) has rapidly increased over the last decade due to their improved pharmacokinetic properties, efficacy, and safety compared with oral vitamin K antagonists.^1^^–^^4^ As a result, the number of patients requiring treatment for life-threatening bleeding associated with FXa-I use has also increased.^5^ Prior to the availability of a specific reversal agent for bleeding secondary to the FXa-I, these bleeds were treated with 4-factor prothrombin complex concentrates (4F-PCC) containing coagulation factors II, VII, IX and X,^6^^,^^7^ but because patients treated with the FXa-I are not deficient in these coagulation factors, the mechanistic rationale for their use has been questioned.^8^ Andexanet alfa (more recently known as coagulation factor Xa [recombinant], inactivated-zhzo) is a specifically designed recombinant factor Xa decoy molecule.^9^ Due to its high affinity to the FXa-I, andexanet alfa binds with apixaban and rivaroxaban releasing native FXa and resulting in thrombin generation, clot formation, and hemostasis.

The efficacy and safety of andexanet alfa in patients treated with FXa-I presenting with life-threatening bleeds is supported by the ANNEXA-4 clinical trial.^10^ However, ANNEXA-4 was a single arm trial. An ongoing randomized clinical trial (RCT), ANNEXA-I, is comparing andexanet alfa head to head with coagulation factor replacement strategy (anticipated to mostly be 4F-PCC) for factor FXa-I-related intracerebral hemorrhage (ICH) (ClinicalTrials.gov NCT03661528). Yet the results of this trial are not anticipated for at least 1–2 years, and it will only provide results for intracerebral bleeds.

While a number of studies have described the use of 4F-PCC for treatment of life-threatening bleeds in patients treated with a FXa-I,^6^^,^^7^ relatively few studies have compared 4F-PCC to andexanet alfa and none have been RCTs.^11^^–^^16^ Direct comparison of patients treated with 4F-PCC and andexanet alfa is challenging due to differences in patient populations and endpoints. A retrospective review of electronic health records (EHR) from 45 hospitals compared outcomes of 3,030 patients hospitalized with major bleeding related to FXa-I and treated with andexanet alfa or 4F-PCC. In this study, treatment with andexanet alfa was associated with the lowest hospital mortality across different bleed types.^14^ However, this study did not collect critical baseline information such as time from last FXa-I dose and did not control for confounding variables, limiting its conclusions. Furthermore, this study was limited to hospital outcomes only with no longer term outcomes.

Another retrospective study of 322 patients treated with andexanet alfa from the ANNEXA-4 study^10^ that were propensity score matched (PSM) with 88 patients from the ORANGE study^15^ found lower 30-day adjusted mortality rates in patients treated with andexanet alfa.^13^ This study is also limited since patients included in the ANNEXA-4 study were generally less severely ill than those included in the ORANGE study. In contrast, other retrospective comparisons of patients treated with andexanet alfa or 4F-PCC have not shown clear differences in outcomes.^11^^,^^12^^,^^16^^,^^17^ Importantly, these studies often failed to control their analyses for relevant patient characteristics that are potential confounding variables, such as timing from last FXa-I dose, timing from onset of bleeding episode, initial ICH volume, time from ICH onset to first head CT, or severity of illness.^18^

Our objective in this current feasibility study was to conduct a multicenter retrospective chart review to determine whether data regarding important patient characteristics was generally documented in the EHR and to explore the association between 4F-PCC or andexanet alfa and outcomes in patients with primary ICH and gastrointestinal bleeds (GIB) associated with use of apixaban or rivaroxaban.

## METHODS

### Study Design

We performed a structured, retrospective chart review, consistent with the recommended methodology of Kaji et al.^19^ Our study also followed the Strengthening of Reporting of Observational Studies in Epidemiology (STROBE) reporting guidelines for cross-sectional studies.^20^ Because of the retrospective design, we received institutional review board (IRB) approval with waiver of informed consent. Due to the nature of this study as a feasibility and exploratory pilot, no sample size calculations were made.

### Patients and Settings

We included all adult patients presenting with an ICH (50) or GIB (50) event who received reversal with andexanet alfa (50) or replacement with 4F-PCC (50). We chose to include 50 patients in each treatment group and bleeding type a priori since we felt that this number would be adequate to determine the feasibility of a larger study. We excluded patients who were treated with both agents as well as patients treated with andexanet alfa plus other coagulation factor concentrates. We also excluded patients transferred to one of the study sites from another hospital. The study period was from May 2018 (when the US Food and Drug Administration [FDA] approved andexanet alfa) until December 2021. Consecutive patients were enrolled in reverse chronological order starting with the most recent date (based on each institution’s IRB approval) until the required number of patients were identified. The study sites included two large academic medical centers and two large community settings. Initially, each hospital was requested to contribute cases equally. However, due to imbalances in number of patients treated, the final number of study patients from each site was not equal. One site did not have andexanet alfa on formulary and only contributed cases treated with 4F-PCC. At the other three sites andexanet alfa was added to the formulary in June 2018.

Decision on high or low dose followed the standard recommendations. No patient in the study had repeated doses. Andexanet alfa was used in patients who reported last dose of apixaban or rivaroxaban within 18 hours and met the following indications: i) acute, overt major or life-threatening bleeding episode; ii) acute bleeding in a critical area or organ, such as pericardial, intracranial, or intraspinal; iii) signs or symptoms of significant hemodynamic compromise despite aggressive fluid and blood product resuscitation; iv) patients with ICH must have had a Glasgow Coma Score (GCS) ≥7 and an estimated intracerebral hematoma volume of 60 milliliters as assessed by computed tomography (CT) or magnetic resonance imaging. During the study period, prescription of andexanet alfa was restricted to neurology, neurosurgery, hematology, emergency medicine, and trauma surgery. The 4F-PCC prescription was not restricted by specialty, and dose was weight-based. The decision to treat patients and the selection of the reversal agent was at the discretion of the treating physician.

### Data Collection

Structured data collection was performed by physicians, pharmacists or trained research assistants using a HIPPA-compliant encrypted database (REDCap, hosted at Vanderbilt University, Nashville, TN). We developed a library of the definitions of data collected. Data collected included demographic, clinical, laboratory, and imaging data on patient presentation and throughout their entire hospital admission and extending to 90 days after discharge. The presence of comorbidities was determined by chart review. Among the variables collected we specifically searched all EHRs for time of bleeding onset, time from last dose of oral FXa-I, time from presentation to imaging, clinical risk scores (GCS for ICH and albumin level <3.0 grams per deciliter, international normalized ratio [INR] >1.5, altered mental status, systolic blood pressure less than 90 millimeters of mercury, and age >65 years [AIMS65] for GIB)^21^^,^^22^ and concomitant treatments including blood products, reversal agents, and procedures. (See Supplements 1–3 for data collection tools.) Thrombotic events were collected during index hospitalization and at 30 and 90 days after discharge. We defined thrombotic events as deep vein thrombosis (DVT), pulmonary embolism (PE), ischemic stroke, myocardial infarction, or other arterial or venous thromboembolic events. Door to needle time was defined as time from hospital presentation to receipt of reversal agent. Onset to CT time was calculated using the difference between initial CT and time of bleeding onset, where they both existed; onset to CT time for the remainder of cases was determined by combining categorical onset to presentation time (<6, 6–12, 12–24, 24–48, and >48 hours), using the median time for each category, with door to CT time. We defined all study data and variables prior to initiating the study and trained our data abstractors using a library of definitions. We periodically monitored data collection and provided feedback to the data abstractors during and after data collection and entry regarding missing, conflicting, or obviously erroneous data. The number of data abstractors at each institution varied from 1–3. The data abstracters were not blinded to therapy.

### Outcomes

We developed all study outcomes a priori. The primary outcomes were presence of estimated times from last dose of FXa-I and time from bleeding onset to administration of 4F-PCC or andexanet alfa. Secondary outcomes were hemostatic efficacy as defined by the ANEXXA-4 criteria,^23^ survival to hospital discharge, thrombotic events during the index hospitalization and at 30 and 90 days, and rebleeding events such as ICH, rectal bleeding, melena, or hematemesis.

### Data Analysis

We used descriptive statistics to summarize the data. Categorical data are presented as numbers or percentages and compared between groups using chi-square or Fisher exact tests as appropriate. Continuous variables are presented with means and standard deviations or medians and interquartile ranges (IQR) based on their distribution and compared with *t*-tests or the Mann-Whitney U tests, as appropriate. Because this was a pilot study, no formal sample size calculation was performed. We chose to include 25 patients in each of the four study subgroups: ICH treated with andexanet alfa; ICH treated with 4F-PCC; GIB treated with andexanet alfa, and GIB treated with 4F-PCC. An exploratory PSM model was constructed to estimate the odds of excellent/good hemostatic efficacy in patients treated with andexanet alfa or 4F-PCC adjusting for age, gender, comorbidities, time from last dose of FXa-I, time from bleed onset to treatment, and indication for anticoagulation.

## RESULTS

### General Characteristics and Feasibility

In total, the study enrolled 100 patients who were treated with either andexanet alfa or 4F-PCC for reversal of life-threatening bleeds after taking apixaban or rivaroxaban (Table 1). As intentionally designed, half of the patients had an ICH and half had a GIB. Within the two groups of patients, half were treated with andexanet alfa while the other half were treated with 4F-PCC. Mean (SD) age of all patients was 75 (12) years, and 34% were female. Most patients were on apixaban (72%), and the rest were on rivaroxaban. Common comorbidities included hypertension (78%) and diabetes (25%). Antiplatelet agents were used in about half of the patients, mostly aspirin (40%) followed by clopidogrel (9%) and ticagrelor (2%). Overall hemostatic efficacy was excellent in 62% and good in an additional 16%. Mortality at hospital discharge was 16%, and the overall rates of thrombosis and rebleeding were 15% and 7%, respectively.

**Table 1.** Comparison of patients with intracerebral hemorrhage and gastrointestinal bleeding. Results are presented as numbers (%) unless otherwise specified.

	Number (%) unless otherwise specified		
	All cases (N = 100)	ICH (n = 50)	GIB (n = 50)
Mean (SD) age, years	75 (12)	75 (15)	75 (10)
Gender			
Female	34 (34)	15 (30)	19 (38)
Male	66 (66)	35 (70)	31 (62)
Ethnicity			
Non-Hispanic	95 (95)	48 (96)	47 (94)
Hispanic	3 (3)	1 (2)	2 (4)
Unknown	2 (2)	1 (2)	1 (2)
Race			
White	91 (91)	47 (94)	44 (88)
Black	2 (2)	1 (2)	1 (2)
Asian	2 (2)	0	2 (4)
Native Hawaiian/Pacific Islander	1 (1)	0	1 (2)
Unknown	4 (4)	2 (4)	2 (4)
Mean height (SD), cm^*^	170 (13)	173 (10)	167 (15)
Mean weight (SD), kg	84 (24)	84 (20)	85 (27)
Mean BMI^*^	30 (15)	28 (6)	31 (21)
Code status at presentation			
Full code	62 (62)	31 (62)	31 (62)
DNR/DNI	10 (10)	5 (10)	5 (10)
DNR	2 (2)	2 (4)	0
Unspecified	26 (26)	12 (24)	14 (28)
Comorbidities			
None	3 (3)	0	3 (6)
Hypertension	78 (78)	44 (88)	34 (68)
Diabetes mellitus	25 (25)	11 (22)	14 (28)
Liver	4 (4)	2 (4)	2 (4)
Chronic kidney disease	13 (13)	2 (4)	11 (22)
Alcohol abuse	8 (8)	3 (6)	5 (10)
Prior bleed	16 (16)	3 (6)	13 (26)
Prior stroke	18 (18)	9 (18)	9 (18)
Factor Xa inhibitor			
Apixaban	72 (72)	36 (72)	36 (72)
Rivaroxaban	28 (28)	14 (28)	14 (28)
Bleed type			
Traumatic	5 (5)	4 (8)	1 (2)
Spontaneous	94 (94)	46 (92)	48 (96)
Unspecified	1 (1)	0	1 (2)
Indications for anticoagulation			
Atrial fibrillation	68 (68)	33 (66)	35 (70)
Deep vein thrombosis	28 (28)	15 (30)	13 (26)
Pulmonary embolism	15 (15)	6 (12)	9 (18)
Prophylaxis of VTE	0	0	0
Other	6 (6)	4 (8)	2 (4)
Antiplatelet agents			
None	52 (52)	24 (48)	28 (56)
Aspirin	40 (40)	23 (46)	17 (34)
Clopidogrel	9 (9)	4 (8)	5 (10)
Ticagrelor	2 (2)	1 (2)	1 (2)
Treatments			
Andexanet alfa	50 (5)	25 (50)	25 (50)
4F-PCC	50 (50)	25 (50)	25 (50)
Number of 4F-PCC doses			
1	43 (86)	19 (76)	24 (96)
2	7 (14)	6 (24)	1 (4)
Vitamin K	12 (12)	9 (18)	3 (6)
Fresh frozen plasma	8 (8)	0	8 (16)
Packed red blood cells	38 (38)	0	38 (76)
Platelets	22 (22)	11 (22)	11 (22)
Factor IX	5 (5)	2 (4)	3 (6)
Desmopressin	7 (7)	6 (12)	1 (2)
Cryoprecipitate pooled 5-pack status	1 (1)	0	1 (2)
Intravenous fluids	59 (59)	31 (62)	28 (56)
Other	7 (7)	3 (6)	4 (8)
Outcomes			
Thrombotic eventsin-hospital	11 (11)	5 (10)	6 (12)
Hemostatic efficacy			
Excellent	62 (62)	35 (71)	27 (55)
Good	16 (16)	5 (10)	11 (22)
Poor	20 (20)	9 (18)	11 (22)
Unknown	2 (2)	1 (2)	1 (2)
Rebleed in-hospital	7 (7)	1 (2)	6 (12)
Survival (to discharge)	83 (83)	42 (84)	41 (82)

* 9 cases missing height and body mass index.

*ICH*, intracerebral; *GIB*, gastrointestinal bleed; *BMI*, body mass index; *VTE*, venous thromboembolism; *DNR*, do not resuscitate.

While the exact timing of last dose of FXa-I and bleed onset was rarely available, estimated ranges of time (in 6–12-hour intervals) from last dose and bleed onset were available in the EHR in most cases. Of those with ICH, 96% of patients who received andexanet alfa and 80% of those who received 4F-PCC had documentation of an estimated time from last FXa-I. In patients with GIB, the documentation of last FXa-I was present in 92% of those receiving 4F-PCC and in 68% of those receiving andexanet alfa.

### Intracerebral Hemorrhage

A comparison of baseline characteristics of patients with ICH based on reversal agent is presented in Table 2. Overall, the two study groups were fairly well balanced for the variables measured. More patients in the 4F-PCC group had a prior stroke and intraventricular extension. The most common sites of bleeding were lobar and the deep white matter. Median (IQR) initial GCS in patients treated with andexanet alfa and 4F-PCC were 14 (12–15) and 14 (13–15), respectively.

**Table 2.** Andexanet alfa vs 4F-PCC in patients with intracerebral hemorrhage. Results are presented as numbers (%) unless otherwise specified.

	Andexanet alfa (n = 25)	4F-PCC (n = 25)	*P-*value
Gender			0.54
Female	9 (36)	6 (24)	
Male	16 (64)	19 (76)	
Mean (SD) age, years	77 (12)	73 (17)	0.38
Ethnicity			0.35
Non-Hispanic	23 (92)	25 (100)	
Hispanic	1 (4)	0	
Unknown	1 (4)	0	
Race			0.22
White	23 (92)	24 (96)	
Black	0	1 (4)	
Asian	0	0	
Native Hawaiian/Pacific Islander	0	0	
Unknown	2 (8)	0	
Comorbidities			
None	0	0	-
Hypertension	23 (92)	21 (84)	0.67
Diabetes mellitus	8 (32)	3 (12)	0.17
Liver disease	0	2 (8)	0.49
Chronic kidney disease	1 (4)	1 (4)	1.00
Alcohol abuse	1 (4)	2(8)	1.00
Prior bleed	0	3 (12)	0.24
Prior stroke	1 (4)	8 (32)	0.02
Indications for anticoagulation			
Atrial fibrillation	17 (68)	16 (64)	1.00
Deep vein thrombosis	8 (32)	7 (28)	1.00
Pulmonary embolism	4 (16)	2 (8)	0.67
Other	1 (4)	3 (12)	0.61
ICH location			
Deep white matter	9 (36)	8 (32)	1.00
Lobar	11 (44)	13 (52)	0.78
Brainstem	2 (8)	0	0.49
Cerebellum	3 (12)	3 (12)	1.00
Intraventricular extension	1 (4)	7 (28)	0.049
Hematoma volume, mean (SD), ml	15 (23)	28 (37)	0.07
Estimated time from last dose of apixaban/rivaroxaban to presentation			0.06
<6 hrs.	7 (28)	1 (4)	
7–12 hrs.	13 (52)	11 (44)	
8–18 hrs.	4 (16)	5 (20)	
19–24 hrs.	0	1 (4)	
>24 hrs.	0	2 (8)	
Unknown	1 (4)	5 (20)	
Estimated time from bleed onset to presentation			0.26
<6 hrs.	18 (72)	17 (68)	
7–12 hrs.	1 (4)	4 (16)	
8–18 hrs.	2 (12)	0	
19–24 hrs.	1 (4)	2 (8)	
>24 hrs.	1 (4)	2 (8)	
Unknown	1 (4)	0	
Median (IQR) GCS	14 (12–15)	14 (13–15)	0.40
Door to CT time (median ([IQR]), hrs.	0.45 (0.22–1.22)	0.53 (0.26–1.16)	0.89
Estimated time from bleed onset to CT*, (median [IQR]), hrs.	4.5 (3.2–6.9)	2.5 (1.1–7.2)	0.25
Door to needle time, median (IQR), hrs.	1.8 (1.4–3.0)	1.7 (1.5–2.6)	0.99
Outcomes			
Thrombotic event inpatient	3 (12)	2 (8)	1.00
Hemostatic efficacy			0.50
Excellent	20 (80)	15 (60)	
Good	2 (8)	3 (12)	
Poor	3 (12)	6 (24)	
Unknown	0	1 (4)	
Grouped hemostatic efficiency			0.29
Excellent/Good	22 (88)	18 (76)	
Fair/Poor	3 (12)	6 (24)	
Rebleed	0	1 (4)	1.00
Survival (to hospital discharge)	23 (92)	19 (76)	0.25
Interventions			
Endotracheal intubation	2 (8)	11 (44)	0.10
Craniotomy	0	8 (32)	0.11
External ventricular drain	0	3 (12)	0.24
Diagnostic angiography	1 (4)	0	
Angiographic repair	0	1(4)	

* Estimated time from bleed onset to CT imaging was calculated by adding the median of the estimated time interval from bleed onset to presentation to the time from door to CT imaging.

*ICH*, intracerebral; *IQR*, interquartile range; *CT*, computed tomography.

In both groups, over two thirds of patients presented within six hours of the onset of bleeding. The proportion of patients presenting within six and within 12 hours of their last dose of oral Fxa-I among those treated with andexanet alfa were 28% and 52%, respectively, as compared with 4% and 44% among those treated with 4F-PCC (*P* = 0.06). Initial hematoma volumes in patients treated with andexanet alfa and 4F-PCC were 15 +/−23 ml and 28+/−37 ml, respectively, *P* = 0.07. Time to imaging and time to administration of the reversal agent were relatively short and comparable.

Hemostatic efficacy was excellent in 80% of those treated with andexanet alfa and 60% among patients treated with 4F-PCC. Survival to hospital discharge among those treated with andexanet alfa and 4FPCC were 92% and 76%, respectively (*P* = 0.25). Of the five thrombotic events that occurred in-hospital, three were in patients treated with andexanet alfa (one ischemic stroke, one DVT, and one PE), and two were in patients treated with 4F-PCC (two DVTs). The rate of additional thrombotic events beyond the index hospitalization among patients treated with andexanet alfa and 4F-PCC were 0% and 5% at 30 days and 0% and 5% at 90 days, respectively.

### Gastrointestinal Bleeding

Of 50 patients with GIB, 25 (50%) had an upper source of GIB, 16 (32%) had a lower source of GIB and in nine (18%) patients, the source was unknown. A comparison of baseline characteristics of patients with GIB based on treatment strategy is presented in Table 3. Overall, the two study groups were fairly well balanced for the variables that were measured. Median (IQR) initial AIMS65 scores in patients treated with andexanet alfa and 4F-PCC were 2 (1–2) and 2 (2–2), respectively. Most patient in both groups had hypertension or diabetes.

**Table 3.** Andexanet alfa v. 4F-PCC in patients with gastrointestinal bleeding. Results are presented as numbers (%) unless otherwise specified.

	Andexanet alfa (n = 25)	4F-PCC (n = 25)	*P*-value
Gender			1.00
Female	9 (36)	10 (40)	
Male	16 (64)	15 (60)	
Mean (SD) age, years	72 (11)	78 (9)	0.049
Ethnicity			0.22
Non-Hispanic	23 (92)	24 (96)	
Hispanic	2 (8)	0	
Unknown	0	1 (4)	
Race			0.39
White	21 (84)	23 (92)	
Black	0	1 (4)	
Asian	2 (8)	0	
Native Hawaiian/Pacific Islander	1 (4)	0	
Unknown	1 (4)	1 (4)	
AIM65 criteria			
Albumin < 3 g/dL	10 (48)	10 (42)	0.77
INR > 1.5	10 (43)	9 (39)	1.00
Altered mental status	6 (24)	4 (17)	0.73
SBP < 90 mm Hg	9 (36)	7 (29)	0.76
Age > 65 years	19 (76)	23 (92)	0.25
Median (IQR) AIMS65 score	2 (1–2)	2 (2–2)	0.90
Comorbidities			
None	2 (8)	1 (4)	1.00
Hypertension	16 (64)	18 (72)	0.76
Diabetes mellitus	7 (28)	7 (28)	1.00
Liver disease	0	2 (8)	0.49
Chronic kidney disease	6 (24)	5 (20)	1.00
Alcohol abuse	2 (8)	3 (12)	1.00
Prior bleed	5 (20)	8 (32)	0.52
Prior stroke	4 (16)	5 (20)	1.00
Indications for anticoagulation			
Atrial fibrillation	15 (60)	20 (80)	0.22
Deep vein thrombosis	6 (24)	7 (28)	1.00
Pulmonary embolism	5 (20)	4 (16)	1.00
Other	2 (8)	0	0.49
Estimated time from last dose of apixaban/rivaroxaban to treatment			<0.001
<6 hrs.	4 (16)	9 (36)	
7–12 hrs.	1 (4)	6 (24)	
8–18 hrs.	3 (12)	7 (28)	
19–24 hrs.	1 (4)	1 (4)	
Unknown	16 (64)	2 (8)	
Estimated time from bleed onset to presentation			0.10
<6 hrs.	10 (40)	13 (52)	
7–12 hrs.	0	2 (8)	
8–18 hrs.	0	3 (12)	
19–24 hrs.	3 (12)	2 (8)	
>24 hrs.	4 (16)	3 (12)	
Unknown	6 (32)	2 (8)	
Door to needle time, median (IQR)	3.6 (2.2–16.0)	3.3 (1.3–7.0)	0.22
Outcomes			
Thrombotic event in-hospital	4 (16)	2 (8)	0.67
Hemostatic efficacy			0.40
Excellent	16 (64)	11 (44)	
Good	4 (16)	7 (28)	
Poor	5 (20)	6 (24)	
Unknown	0	1 (4)	
Grouped hemostatic efficiency			0.74
Excellent/Good	20 (80)	18 (76)	
Fair/Poor	5 (20)	6 (24)	
Rebleed in-hospital	5 (20)	1 (4)	0.09
Survival (to hospital discharge)	19 (76)	22 (88)	0.46
Interventions			
Mean (SD) units PRBC over 24 hours	1.6 (1.8)	2.2 (1.6)	0.26
Endoscopy	14 (56)	21 (84)	0.06
Interventional radiology	1 (4)	2 (8)	1.00
Surgery	2 (8)	1 (4)	1.00

*INR*, International normalized ratio; *SBP*, systolic blood pressure; *IQR*, interquartile range; *PRBC*, packed red blood cells.

The percentages of patients presenting within 6 or 12 hours of bleeding onset among those treated with andexanet alfa were 40% and 0%, while the percentages of patients presenting within 6 or 12 hours of bleeding were 52% and 8%, respectively, among patients treated with 4F-PCC. The time from bleeding onset to presentation was greater than 24 hours or unknown in 16% and 32% in patients treated with andexanet alfa and 12% and 8% in patients treated with 4F-PCC, respectively. The time of the last dose of Fxa-I was 12 hours or less in 20% and 60% in patients treated with andexanet alfa and 4F-PCC, respectively. Median [IQR] times from arrival to ED to administration of the treatment drug were also relatively short and comparable in patients treated with andexanet alfa and 4F-PCC (3.6 [2.2–16.0] vs 3.3 [1.3–7.0], respectively).

Hemostatic efficacy was excellent in 64% and 44% of those treated with andexanet alfa and 4F-PCC, respectively (*P* = 0.40). Survival to hospital discharge among those treated with andexanet alfa and 4F-PCC were 76% and 88%, respectively (*P* = 0.46). The rate of thrombotic events among patients treated with andexanet alfa and 4F-PCC were 8% in each group. Of the six thrombotic events that occurred in-hospital, four were in patients treated with andexanet alfa (two DVTs, and two other) and two were in patients treated with 4F-PCC (two ischemic strokes).

### Thrombotic Events

There were 15 cases with recorded thrombotic events (Tables 4 and 5). Of these, 11 cases had in-hospital thrombotic events, three had thrombotic events at 30 days, and one event occurred between 30-90 days of index bleed. Of all thrombotic events, seven occurred in patients treated with andexanet alfa and eight occurred in patients treated with 4F-PCC. Of the 15 patients who had a thrombotic event 10 patients were restarted on anticoagulation prior to the thrombotic event. A breakdown of thrombotic events by site of bleeding and therapy is presented in Table 5.

**Table 4.** Outcomes by treatment when bleeding types are combined. Numbers (percent).

	Andexanet alfa (n = 50)	4F-PCC (n = 50)	*P*-value
Hemostatic efficacy			0.26
Excellent	36 (72)	26 (52)	
Good	6 (12)	10 (20)	
Poor	8 (16)	12 (24)	
Grouped hemostatic efficiency			0.32
Excellent/good	42 (84)	36 (72)	
Fair/poor	8 (16)	12 (24)	
Rebleeding events in hospital	5 (10)	2 (4)	0.26
Survival to hospital discharge	42 (84)	41 (82)	1.00
In-hospital thrombotic events	7 (14)	4 (8)	0.53
Total thrombotic events through day 90	7 (14)	8 (16)	0.80

**Table 5.** Summary of thrombotic events.

	Bleed site	In-hospital event	30 days	90 days
Andexanet alfa	ICH	3 (1 DVT, 1 stroke, 1 PE)	0	0
	GIB	4 (2 DVT, 2 other^*^)	0	0
4F-PCC	ICH	2 (2 DVT)	1 (DVT)	1 (DVT)
	GIB	2 (2 strokes)	2 (2 DVT)	

* Acute left lower extremity arterial thrombosis with ischemia (n = 1); bowel infarction and splenic infarcts (n = 1).

*ICH*, intracerebral hemorrhage; *DVT*, deep vein thrombosis; *PE*, pulmonary embolism; *GIB*, gastrointestinal bleeding.

### Propensity Score Matched Model

Outcomes in patients treated with andexanet alfa and 4F-PCC when all study patients were compared regardless of site of bleeding are summarized in Table 4. There were no statistically significant differences in any of the study outcomes between the two treatment groups. Using all cases combined (98, excludes two missing hemostatic efficacy) and for the endpoint of hemostatic efficacy, categorized into excellent/good and poor, a propensity score was determined using age, gender, comorbidities, time from last dose, time from bleeding onset, and indication for anticoagulation. Inclusion of the propensity score and treatment in a logistic regression model resulted in an odds ratio (OR) in favor of andexanet alfa of 2.01 (95% confidence interval CI, 0.67–6.06) for the endpoint of excellent/good hemostatic efficacy.

### Additional Exploratory Analyses for Hemostatic Efficacy

For the 49 ICH cases (one with missing data excluded) and for the outcome of hemostatic efficacy, categorized into excellent/good and poor, a propensity score was determined using age, hematoma volume categorized into <30 vs ≥30 milliliters, GCS score categorized into <13 vs ≥13, time from last dose of FXa-I, and onset to CT time. Two cases were excluded (both 4F-PCC) because one did not have an initial CT time and one had initial CT time prior to hospital presentation. Inclusion of the propensity score and treatment in a logistic regression model resulted in an OR of andexanet alfa to 4F-PCC of 3.30 (95% CI 0.59–18.52) in predicting excellent/good hemostatic efficacy.

For 49 GIB cases (one with missing data excluded) and for the outcome of hemostatic efficacy, categorized into excellent/good and poor, a propensity score was determined using age, time from last FXa-I dose, time of bleed onset, and AIMS65 categorized into <2 vs ≥2. Inclusion of the propensity score and treatment in a logistic regression model resulted in an OR of andexanet alfa to 4F-PCC of 1.67 (95% CI 0.29–9.71) in predicting excellent/good hemostatic efficacy.

## DISCUSSION

Our multicenter study demonstrates that a comparative study of patients with ICH and GIB treated with andexanet alfa or 4F-PCC can be adequately conducted by collecting and analyzing data routinely available in the EHR retrospectively. We were able to acquire information on many important factors affecting outcomes, including several that had been consistently missing from previous studies.^18^ For example, documentation of estimated ranges of time from last FXa-I does were present in 80–96% of patients with ICH and in 68%–92% of patients with GIB. These results suggest that a reliable study evaluating real-life practice in patients undergoing FXa-I reversal with andexanet alfa or 4F-PCC after ICH or GIB should be feasible in a much larger cohort of patients in centers like those included in the study.

Current literature on reversal strategies for FXa-I has major limitations. There have been only non-randomized cohort studies evaluating andexanet alfa and 4F-PCC.^24^^–^^26^ Most of these studies have been single-arm, single-center, or both.^6^^,^^7^^,^^11^^,^^12^^,^^16^^,^^27^^–^^35^ Some use historical rather than contemporary controls, or at best indirectly compared two independent datasets trying to account for major baseline differences by using a suboptimal PSM.^13^ Other studies used propensity score treatment weighting rather than PSM and were able to account for bleed size/volume, which was not captured in the ORANGE indirect comparison.^36^^,^^37^ None has included all pertinent variables to sufficiently reduce the risk of confounding bias.^18^ Even ANNEXA-4,^10^^,^^23^ a prospective study that served to support the FDA approval of andexanet alfa for reversal of anticoagulation with FXa-I in patients with life-threatening bleeding, had a single arm and failed to control for crucial factors, such as time from ICH onset to first head CT— a key variable because the risk of hematoma expansion is much greater in patients presenting early after ICH onset.^38^ Other pertinent variables often missed in most previous studies include time between ICH onset and hospital presentation, time between last dose of FXa-I and hospital presentation, precise hematoma volume and location, measures of severity of GIB, concomitant administration of antiplatelet agents, detailed accounting of comorbidities, restrictions in the level of medical care, and functional outcomes.^18^ Up-to-date assays to measure level of anticoagulation in patients treated with FXa-I are not commercially available. In the absence of information on the intensity of anticoagulation, time variables (eg, time from last dose of anticoagulant) become key surrogates in a controlled analysis of hemostatic efficacy.

One of the major advantages of RCTs is that the act of randomization reduces the chance of between-group bias due to imbalances in measured and unmeasured confounding variables. Thus, any claims regarding the relative efficacy and safety of andexanet alfa vs 4F- PCC in patients with FXa-I bleeds will only be resolved with the completion of a RCT comparing these treatments head to head. That said, in the meantime real-world data can be useful if the studies adjust as much as possible for potential confounding variables.

Our four-center study was designed to determine the feasibility of collecting all relevant information from the EHR of multiple centers. Due to the small sample size, adjustment of covariates in a multivariate analysis was limited, and no definitive conclusions can be made regarding the relative efficacy and safety of andexanet alfa and 4F-PCC. Our exploratory analysis including PSM models suggested better hemostatic efficacy with andexanet alfa for both indications, ICH and GIB. However, these results were very imprecise (as indicated by the wide 95% CIs) and should therefore be interpreted with great caution.

The strength of our study resides in the granularity of the data collection. Among patients with ICH, we were able to obtain information on time between last dose of FXa-I and symptom onset and the intervals between symptom onset and hospital presentation and first head CT. While exact times were not consistently documented (in fact, this information is often not known with precision in daily practice), estimated time ranges were available in nearly all patients. Clinical severity assessed by the GCS score and the use of concomitant antiplatelet agents were routinely available. Intracerebral hematoma volumes were also available since all patients with ICH had initial head CTs as well as repeat CTs within 6–24 hours, allowing precise calculation of hematoma volume and hematoma expansion. For patients with GIB, we were able to adjust for illness severity using the AIMS65 risk assessment score. The hemostatic efficacy in patients with GIB is typically determined by the need for blood transfusions and their effect on hemoglobin and hematocrit over the course of the first 12 hours. However, bleeding cessation often requires advanced interventions, such as endoscopic hemostasis or embolization by interventional radiology. Data on these advanced interventions are also important to report; however, many prior studies did not report on these interventions.

Of note, the mortality in the subgroup of patients with GIB in this study was considerably higher than previously reported,^39^ suggesting that patients with GIB while taking FXa-I are a particularly high-risk group and require immediate and aggressive therapy. Existing risk prediction scores, such as the AIMS65,^40^ do not account for ongoing use of FXa-I, yet this factor must be considered in the management patients with GIB. Due to the lack of RCTs and the small number of reported studies evaluating use of andexanet alfa for acute GIB the American College of Gastroenterology and the Canadian Association of Gastroenterology recommend against the use of andexanet alfa for reversing life-threatening gastrointestinal bleeds.^35^ More data is needed to identify the ideal patient and reversal agent for FXa-I-associated GIB.

## LIMITATIONS

This study has various limitations in addition to the small size of the four groups examined. Selection bias may have affected our findings, and the direction of this bias is difficult to infer from our data. In institutions where both andexanet alfa and 4-PCC are available, the treated groups become selectively different and so is their prognosis. For example, because of stricter criteria for the use of andexanet alfa the use of 4-PCC may be relegated to patients with worse bleeds and less favorable chances of recovery. On the other hand, andexanet alfa could have been preferentially used in patients deemed to be at higher risk of hematoma expansion. Thus, confounding by indication cannot be excluded. Studying a much larger cohort with adequate adjustment for covariates might be able to account for these issues.

Also, functional outcomes after discharge were available in only some of the participating centers. Information on thrombotic events was consistently available in the hospital but not after discharge. One of the participating centers did not have availability of andexanet alfa and consequently the other three centers had to contribute more cases treated with this agent. This may have introduced additional bias to our study. Identification of thrombotic events and deaths that occurred after hospital discharge may have been underestimated since some patients may have followed up at hospital sites other than the index hospital.

Due to the nature of the study being a feasibility pilot, data abstraction was performed by investigators who were not blinded to therapy. This may have introduced observer bias that may have affected the exploratory results. While overall documentation of important confounding variables at the four study sites was good to excellent, it is possible that other sites may have had less (or more) documentation of these variables. In this study we chose the AIMS65 score to control for gastrointestinal bleeding severity. We acknowledge that the AIMS65 has not been validated in lower GIB and may not be the best index of severity even in upper GIB. In addition, since the median AIMS65 score was relatively low in both study groups, our results may not be generalizable to more severe gastrointestinal bleeds.

Another limitation of our study is that it included mostly White patients and may not be as representative of other racial minority groups. While none of the patients received more than one dose of andexanet alfa, some of the patients received a second dose of 4F-PCC. However, it is unclear why a second dose was given. Finally, we have no information regarding compliance with FXa-I after discharge from the hospital, which may contribute to a high rate of thrombotic events.

## CONCLUSION

This feasibility study indicates that careful collection of information routinely available in the electronic health records of academic and community hospitals permits the development of adjustment models incorporating most of the factors that can influence the prognosis of patients with intracranial hemorrhage and gastrointestinal bleeds and confound the interpretation of the therapeutic effects of anticoagulation reversal with andexanet alfa or replacement with 4F-PCC. Until more definitive data from randomized controlled trials becomes available, a much larger study with more participating centers may produce comparative real-life experience that can help determine whether the treatment selected for reversal of FXa-I (ie, andexanet alfa vs 4F-PCC) has an impact on patient-centered outcomes in daily practice. Furthermore, our data has additional benefit since it reflects clinical practice where patients are not necessarily treated as in well controlled trials.

## Supplementary Information






