# Supplementary material for: Treatment of Factor-Xa Inhibitor-associated Bleeding with Andexanet Alfa or 4 Factor PCC: A Multicenter Feasibility Retrospective Study
Source: West J Emerg Med. 2023 Aug 22;24(5):939–49. doi: 10.5811/westjem.60587 (PMC10527834; doi:10.5811/westjem.60587)
Supplement: Supplementary file 1 [file wjem-24-939-s001.pdf]

Comparison of 4-Factor PCC and Andexxa for Factor XA inhibitor associated Bleeding

Record ID	________________ (auto-completed by REDCap)
Name of Reviewer	________________
Age	________________ (Year)
Sex	◯ Female<br>◯ Male
Ethnicity	◯ Hispanic or Latino<br>◯ NOT Hispanic or Latino<br>◯ Unknown / Not Reported
Race	◯ American Indian/Native American<br>◯ Asian<br>◯ Native Hawaiian or Other Pacific Islander<br>◯ Black or African American<br>◯ White<br>◯ More than One Race<br>◯ Unknown / Not Reported
Height	________________ (cm)
Weight	________________ (kg)
BMI	________________
Mode of Presentation to Hospital	◯ ED Presentation<br>◯ Inter-Facility Transfer
Presentation to Hospital	________________
Onset of Bleeding Event	________________
Onset to Presentation Difference	________________

Co-morbidities

- ☐ No significant PMH
- ☐ HTN
- ☐ DM
- ☐ Liver Disease
- ☐ CKD
- ☐ Alcohol Abuse
- ☐ Prior bleeding episodes
- ☐ Prior stroke

Antithrombotic agent

- ◯ None
- ◯ Apixaban
- ◯ Rivaroxaban

Home Dose of Antithrombotic Agent

____
(mg)

Indication(s) for anticoagulation:

- ☐ A-fib
- ☐ DVT
- ☐ PE
- ☐ Prophylaxis of VTE
- ☐ Mitral Valve Replacement
- ☐ ECMO
- ☐ Other

If other, please describe

____

Date & Time of last anticoagulant dose (if known)

____

Antiplatelet Agents:

- ☐ None
- ☐ Aspirin
- ☐ Clopidogrel
- ☐ Ticagrelor
- ☐ Other

Specify other antiplatelet agent

____

Date & Time of last antiplatelet dose (if known)

____

Presentation Details

Bleed Type

- ◯ Traumatic
- ◯ Spontaneous

Site of Bleed

- ☐ ICH
- ☐ GI Bleed
- ☐ Other

If ICH, please specify

- ☐ SAH
- ☐ Intraparenchymal
- ☐ Intraventricular
- ☐ SDH
- ☐ EDH

If ICH, initial hematoma volume

____
(mL)

Modified Rankin Scale upon Presentation

____

NIHSS upon Presentation

____

GCS upon Presentation

____

ICH Score (from CT)

____

IF GI Bleed, please specify:

- ☐ Upper
- ☐ Lower
- ☐ Unknown

If Other, please specify:

- ☐ Epistaxis
- ☐ Vaginal bleeding
- ☐ Retroperitoneal
- ☐ Pericaridal
- ☐ Intrathoracic
- ☐ Intraperitoneal
- ☐ AAA
- ☐ Extremities
- ☐ Hematuria
- ☐ Other

Bleed description

____

Was an Anti-Xa level obtained?

- ◯ Yes
- ◯ No

If yes, indicate date and time

____

If yes, please describe

- ◯ Detected
- ◯ Not Detected

Was a Factor Xa level obtained?

- ◯ Yes
- ◯ No

If yes, indicate date & time

____

If yes, please describe:

- ◯ Detected
- ◯ Not Detected

Measures Immediately Prior to Reversal

Date/Time of HR Prior to Reversal Agent

____

HR Prior to Reversal Agent

____
(beats per min)

Date/Time of SBP Prior to Reversal Agent

____

SBP Prior to Reversal Agent

____
(mmHg)

Date/Time of DBP Prior to Reversal Agent

____

DBP Prior to Reversal Agent

____
(mmHg)

Date/Time of Hb Prior to Reversal Agent

____

Hb Prior to Reversal Agent

____

Date/Time of Hct Prior to Reversal Agent

____

Hct Prior to Reversal Agent

____

Date/Time of Creatinine Prior to Reversal Agent

____

Creatinine Prior to Reversal Agent

____

Date/Time of PT Prior to Reversal Agent

____

PT Prior to Reversal Agent

____

Date/Time of PTT Prior to Reversal Agent

____

PTT Prior to Reversal Agent

____

Date/Time of INR Prior to Reversal Agent

____

INR Prior to Reversal Agent

____
