# Supplementary material for: Treatment of Factor-Xa Inhibitor-associated Bleeding with Andexanet Alfa or 4 Factor PCC: A Multicenter Feasibility Retrospective Study
Source: West J Emerg Med. 2023 Aug 22;24(5):939–49. doi: 10.5811/westjem.60587 (PMC10527834; doi:10.5811/westjem.60587)
Supplement: Supplementary file 2 [file wjem-24-939-s002.pdf]

Comparison of 4-Factor PCC and Andexxa for Factor XA inhibitor associated Bleeding

Record ID	______________________ (auto-completed by REDCap)
Treatment(s) within 24 Hours of Presentation	☐ Andexxa<br>☐ 4F-PCC (KCentra)<br>☐ 3F-PCC<br>☐ Vitamin K<br>☐ FFP<br>☐ PRBC<br>☐ Platelets<br>☐ Factor IX<br>☐ Factor VII<br>☐ Desmopressin<br>☐ Cryoprecipitate Pooled 5-Pack Status<br>☐ TXA<br>☐ IV Fluids<br>☐ Other<br>(For all agents, please select only the amount of units ADMINISTERED & not ordered)

Andexxa

Andexxa: Number of Treatments	◯ 1<br>◯ 2<br>◯ 3
Andexxa: Method of Dosage	☐ Bolus Dose<br>☐ Infusion Dose
Date/Time of Bolus Dose 1	______________________
Mg of Bolus Dose 1	______________________
Date/Time of Infusion Dose 1	______________________
Mg of Infusion Dose 1	______________________
Andexxa 2: Method of Dosage	☐ Bolus Dose<br>☐ Infusion Dose
Date/Time of Bolus Dose 2	______________________
Mg of Bolus Dose 2	______________________
Date/Time of Infusion Dose 2	______________________

Mg of Infusion Dose 2	__________________________________
Andexxa 3: Method of Dosage	☐ Bolus Dose<br>☐ Infusion Dose
Date/Time of Bolus Dose 3	__________________________________
Mg of Bolus Dose 3	__________________________________
Date/Time of Infusion Dose 3	__________________________________
Mg of Infusion Dose 3	__________________________________

4-PCC

4-PCC: Number of treatments	◯ 1<br>◯ 2<br>◯ 3<br>◯ 4<br>◯ 5<br>◯ 6
4-PCC 1: Date and time	__________________________________
4-PCC 1: Dose	__________________________________
4-PCC 2: Date and time	__________________________________
4-PCC 2: Dose	__________________________________
4-PCC 3: Date and time	__________________________________
4-PCC 3: Dose	__________________________________
4-PCC 4: Date and time	__________________________________
4-PCC 4: Dose	__________________________________
4-PCC 5: Date and time	__________________________________
4-PCC 5: Dose	__________________________________

4-PCC 6: Date and time

____

4-PCC 6: Dose

____

3F-PCC

3F-PCC: Number of Treatments

- ◯ 1
- ◯ 2
- ◯ 3
- ◯ 4
- ◯ 5

Date & Time of 3F-PCC Treatment

____

3F-PCC: Total number of Units

____
(Units)

3F-PCC 2: Date & Time

____

3F-PCC 2: Dose

____
(Units)

3F-PCC 3: Date & Time

____

3F-PCC 3: Dose

____
(Units)

3F-PCC 4: Date & Time

____

3F-PCC 4: Dose

____
(Units)

Vitamin K

Vitamin K: Number of Treatments

- ◯ 1
- ◯ 2
- ◯ 3
- ◯ 4
- ◯ 5

Vitamin K: Date & Time

____

Vitamin K: Dose

____
(mg)

Vitamin K: Route	◯ IV<br>◯ PO
Vitamin K 2: Date & Time	______________________
Vitamin K 2: Dose	______________________<br>(mg)
Vitamin K 2: Route	◯ IV<br>◯ PO
Vitamin K 3: Date & Time	______________________
Vitamin K 3: Dose	______________________<br>(mg)
Vitamin K 3: Route	◯ IV<br>◯ PO
Vitamin K 4: Date & Time	______________________
Vitamin K 4: Dose	______________________<br>(mg)
Vitamin K 4: Route	◯ IV<br>◯ PO
Vitamin K 5: Date & Time	______________________
Vitamin K 5: Dose	______________________<br>(mg)
Vitamin K 5: Route	◯ IV<br>◯ PO

FFP

FFP: Number of Units	◯ 1<br>◯ 2<br>◯ 3<br>◯ 4<br>◯ 5
FFP 1: Date & Time	______________________

FFP 1: Total number of Units

____
(Units)

FFP 2: Date & Time

____

FFP 2: Total number of Units

____
(Units)

FFP 3: Date & Time

____

FFP 3: Total number of Units

____
(Units)

FFP 4: Date & Time

____

FFP 4: Total number of Units

____
(Units)

FFP 5: Date & Time

____

FFP 5: Total number of Units

____
(Units)

PRBC

PRBC: Number of Units

- ◯ 1
- ◯ 2
- ◯ 3
- ◯ 4
- ◯ 5

PRBC: Date & Time

____

PRBC: Total number of units

____
(Units)

PRBC 2: Date & Time

____

PRBC 2: Number of Units

____

PRBC 3: Date & Time

____

PRBC 3: Number of Units ________________________________

PRBC 4: Date & Time ________________________________

PRBC 4: Number of Units ________________________________

PRBC 5: Date & Time ________________________________

PRBC 5: Number of Units ________________________________

Platelets

Platelets: Number of Units
- ◯ 1
- ◯ 2
- ◯ 3
- ◯ 4
- ◯ 5

Platelets: Date & Time ________________________________

Platelets: Total number of units ________________________________
(Units)

Platelets 2: Date & Time ________________________________

Platelet 2: Number of Units ________________________________

Platelets 3: Date & Time ________________________________

Platelet 3: Number of Units ________________________________

Platelets 4: Date & Time ________________________________

Platelet 4: Number of Units ________________________________

Platelets 5: Date & Time ________________________________

Platelet 5: Number of Units ________________________________

Factor IX

Factor IX: Number of Units

◯ 1
◯ 2
◯ 3
◯ 4
◯ 5

Factor IX: Date & Time

____

Factor IX: Total number of units

____
(Units)

Factor IX 2: Date & Time

____

Factor IX 2: Total number of units

____
(Units)

Factor IX 3: Date & Time

____

Factor IX 3: Total number of units

____
(Units)

Factor IX 4: Date & Time

____

Factor IX 4: Total number of units

____
(Units)

Factor IX 5: Date & Time

____

Factor IX 5: Total number of units

____
(Units)

Factor VII

Factor VII: Number of Units

◯ 1
◯ 2
◯ 3
◯ 4
◯ 5

Factor VII: Date & Time

____

Factor VII: Total number of units

____
(Units)

Factor VII 2: Date & Time

____

Factor VII 2: Total number of units

____

Factor VII 3: Date & Time

____

Factor VII 3: Total number of units

____

Factor VII 4: Date & Time

____

Factor VII 4: Total number of units

____

Factor VII 5: Date & Time

____

Factor VII 5: Total number of units

____

Cryoprecipitate

Cyroprecipitate: Total number of Units

____

Cyroprecipitate: Date & Time

____

Cryoprecipitate: Total number of units

____

Cyroprecipitate 2: Date & Time

____

Cryoprecipitate 2: Total number of units

____

Cyroprecipitate 3: Date & Time

____

Cryoprecipitate 3: Total number of units

____

Cyroprecipitate 4: Date & Time

____

Cryoprecipitate 4: Total number of units

____

Cyroprecipitate 5: Date & Time

____

Cryoprecipitate 5: Total number of units

____

Desmopressin

Desmopressin: Number of Doses

- ○ 1
- ○ 2
- ○ 3
- ○ 4
- ○ 5

Desmopressin: Date & Time

____

Desmopressin: Mg of First Dose

____

Desmopressin 2: Date & Time

____

Desmopressin 2: Mg of Second Dose

____

Desmopressin 3: Date & Time

____

Desmopressin 3: Mg of Third Dose

____

Desmopressin 4: Date & Time

____

Desmopressin 4: Mg of Fourth Dose

____

Desmopressin 5: Date & Time

____

Desmopressin 5: Mg of Fifth Dose

____

TXA

TXA: Number of Doses

- ○ 1
- ○ 2
- ○ 3
- ○ 4
- ○ 5

TXA: Date & Time

____

TXA: Mg of First Dose

____

TXA 2: Date & Time

____

TXA 2: Mg of Second Dose

____

TXA 3: Date & Time

____

TXA 3: Mg of Third Dose

____

TXA 4: Date & Time

____

TXA 4: Mg of Fourth Dose

____

TXA 5: Date & Time

____

TXA 5: Mg of Fourth Dose

____

IV Fluid During First 24 Hours

IV Fluids Date/Time Started

____

Type of IV Fluid

- ☐ Sodium Chloride (Saline)
- ☐ Lactated Ringers
- ☐ 5% Dextrose
- ☐ Other

If other, please specify:

____

IV Fluid Total Volume

____
(mL)

Other treatment

Specify other treatment 1:

____
(Include number of treatments, dose, and method of administration)

Was there a second 'other' treatment?

- ◯ Yes
- ◯ No

Specify other treatment 2:

____
(Include number of treatments, dose, and method of administration)

Interventions

Interventions

- ☐ None
- ☐ Surgery
- ☐ Interventional Radiology
- ☐ Endoscopy
- ☐ Other

Date & Time of Intervention

____

Specify other intervention(s)

____

Which type of surgical procedure?

- ○ Laparotomy
- ○ Craniotomy
- ○ Fasciotomy
- ○ Other

If surgery, describe procedure:

____

Which IR procedure?

- ○ Embolization
- ○ Other

If IR, describe procedure:

____

Which type of endoscopy procedure?

- ○ Injection of sclerotic agent
- ○ Injection of vasoconstrictor
- ○ Banding
- ○ Cauterizing
- ○ Constricting
- ○ Other

If endoscopy, describe procedure:

____

Intervention Description

____

INR Trends

Date/Time 1st INR after reversal agent __________________________________

1st INR after reversal agent __________________________________

Date/Time 2nd INR __________________________________

2nd INR __________________________________

Date/Time of 3rd INR __________________________________

3rd INR __________________________________

Date/Time of 4th INR __________________________________

4th INR __________________________________
