# Supplementary material for: Treatment of Factor-Xa Inhibitor-associated Bleeding with Andexanet Alfa or 4 Factor PCC: A Multicenter Feasibility Retrospective Study
Source: West J Emerg Med. 2023 Aug 22;24(5):939–49. doi: 10.5811/westjem.60587 (PMC10527834; doi:10.5811/westjem.60587)
Supplement: Supplementary file 3 [file wjem-24-939-s003.pdf]

Confidential


# Comparison of 4-Factor PCC and Andexxa for Factor XA inhibitor associated Bleeding

| | |
|---|---|
| Record ID | __________________ (auto-completed by REDCap) |
| If ICH, was a repeat CT/MRI performed? | ◯ Yes<br>◯ No |
| If yes, date & time of repeat CT/MRI | __________________ |
| If yes, please describe volume of hematoma | __________________ |
| Volume Difference | __________________ |
| Hemostatic Efficacy | ◯ Excellent<br>◯ Good<br>◯ Fair<br>◯ Poor |
| Indicate the Modified Rankin Scale (if known) | __________________ |
| NIHSS at Discharge (if known) | __________________ |
| Disposition | ◯ Home from ED<br>◯ Regular Floor<br>◯ ICU<br>◯ N/A, patient expired before leaving ED |
| Date/Time of Disposition | __________________ |
| Was the patient upgraded to the ICU? | ◯ Yes<br>◯ No |
| Date and Time of ICU Upgrade | __________________ |
| Was the ICU upgrade due to a bleeding event? | ◯ Yes<br>◯ No |
| Did the patient restart on any anticoagulation? | ◯ Yes<br>◯ No |
| If yes, date of restart of anticoagulation | __________________ |

| | |
|---|---|
| If yes, which anticoagulant agent? | ☐ Heparin IV<br>☐ Heparin Subcutaneous<br>☐ Coumadin/Warfarin<br>☐ Eliquis/Apixaban<br>☐ Xarelto/Rivaroxaban<br>☐ Dabigatron/Pradaxa<br>☐ Enoxaparin/Lovenox<br>☐ Other |
| If other, please describe | __________________________________ |
| Did the patient develop a thrombotic event while in the hospital? | ◯ Yes<br>◯ No |
| Location of Thrombotic Event | ◯ Regular Floor<br>◯ ICU<br>◯ Other |
| If other, which location | __________________________________ |
| Date/Time of Thrombotic Event in Hospital | __________________________________ |
| What type of thrombotic event occurred in the hospital? | ◯ MI<br>◯ Ischemic Stroke/Stroke<br>◯ TIA<br>◯ DVT<br>◯ PE<br>◯ Other |
| If other, please describe | __________________________________________ |
| Survival to hospital discharge? | ◯ Yes<br>◯ No |
| Date/Time of Hospital Discharge | __________________________________ |
| Hospital length of stay after bleed | __________________________________ |
| ICU Length of Stay from Disposition | __________________________________ |
| ICU Length of Stay from Upgrade | __________________________________ |
| Did the patient develop a thrombotic event within 30 days post-discharge? | ◯ Yes<br>◯ No<br>◯ Unknown |
| If yes, date & time of thrombotic event | __________________________________ |

| | |
|---|---|
| Type of Thrombotic Event | ◯ MI<br>◯ Ischemic Stroke/Stroke<br>◯ TIA<br>◯ DVT<br>◯ PE<br>◯ Other |
| If other, please describe | __________________________________ |
| Based off medical record search, is the patient deceased 30 days after discharge? | ◯ Yes<br>◯ No<br>◯ Unknown |
| If so, cause for death: | ◯ Cardiovascular cause<br>◯ Noncardiovascular cause<br>◯ Uncertain cause |
| Did the patient develop a thrombotic event within 90 days post-discharge? | ◯ Yes<br>◯ No<br>◯ Unknown |
| If yes, date & time of thrombotic event | ____________________________ |
| Type of Thrombotic Event | ◯ MI<br>◯ Ischemic Stroke/Stroke<br>◯ TIA<br>◯ DVT<br>◯ PE<br>◯ Other |
| If other, please describe | __________________________________ |
| Based off medical record search, is the patient deceased 90 days after discharge? | ◯ Yes<br>◯ No<br>◯ Unknown |
| If so, cause for death: | ◯ Cardiovascular cause<br>◯ Noncardiovascular cause<br>◯ Uncertain cause |
| Comments | __________________________________ |
